# Investigation of molecular mechanisms of experimental compounds in murine models of chronic allergic airways disease using synchrotron Fourier-transform infrared microspectroscopy

**DOI:** 10.1038/s41598-020-68671-2

**Published:** 2020-07-16

**Authors:** Nadia Mazarakis, Jitraporn Vongsvivut, Keith R. Bambery, Katherine Ververis, Mark J. Tobin, Simon G. Royce, Chrishan S. Samuel, Kenneth J. Snibson, Paul V. Licciardi, Tom C. Karagiannis

**Affiliations:** 1 Epigenomic Medicine Laboratory, Department of Diabetes, Central Clinical School, Monash University, Alfred Centre, 99 Commercial Road, Melbourne, VIC 3004 Australia; 2 Faculty of Veterinary and Agricultural Sciences, University of Melbourne, Parkville, VIC 3010 Australia; 3 Murdoch Children’s Research Institute, Melbourne, VIC 3004 Australia; 4 ANSTO Australian Synchrotron, Clayton, VIC 3168 Australia; 5 Department of Paediatrics, University of Melbourne, Parkville, VIC 3010 Australia; 6 Department of Pharmacology, Monash Biomedicine Discovery Institute, Monash University, Clayton, VIC 3168 Australia; 7 Department of Clinical Pathology, University of Melbourne, Parkville, VIC 3010 Australia

**Keywords:** Infrared spectroscopy, Asthma

## Abstract

The ovalbumin-induced (OVA) chronic allergic airways murine model is a well-established model for investigating pre-clinical therapies for chronic allergic airways diseases, such as asthma. Here, we examined the effects of several experimental compounds with potential anti-asthmatic effects including resveratrol (RV), relaxin (RLN), l-sulforaphane (LSF), valproic acid (VPA), and trichostatin A (TSA) using both a prevention and reversal model of chronic allergic airways disease. We undertook a novel analytical approach using focal plane array (FPA) and synchrotron Fourier-transform infrared (S-FTIR) microspectroscopic techniques to provide new insights into the mechanisms of action of these experimental compounds. Apart from the typical biological effects, S-FTIR microspectroscopy was able to detect changes in nucleic acids and protein acetylation. Further, we validated the reduction in collagen deposition induced by each experimental compound evaluated. Although this has previously been observed with conventional histological methods, the S-FTIR technique has the advantage of allowing identification of the type of collagen present. More generally, our findings highlight the potential utility of S-FTIR and FPA-FTIR imaging techniques in enabling a better mechanistic understanding of novel asthma therapeutics.

## Introduction

Animal models designed specifically for chronic lung diseases such as asthma are important for investigating the underlying mechanisms of the disease and the effectiveness of potential novel therapies^1^. One of the most widely used models for asthma is the mouse chronic allergic airways disease model that utilises ovalbumin (OVA)^2,3^. This model recapitulates many of the hallmark characteristics of chronic asthma including eosinophilic inflammation, airway hyperresponsiveness, epithelial wall thickening, goblet cell metaplasia, and fibrosis^4,5^.

Current therapies for people with asthma include bronchial dilators, glucocorticosteroids that target inflammation, or a combination of these. However, they have limitations, particularly for patients with severe asthma who are often unresponsive to current treatments^6^. A number of experimental therapies have shown promising beneficial effects in various models of chronic allergic airways diseases^7–10^.

Resveratrol (RV) is a dietary polyphenol found in the skin of red grapes and has been demonstrated to be efficacious in improving airway pathology and function in two different models of chronic allergic airways disease^11,12^. Similarly, l-sulforaphane (LSF), derived from cruciferous vegetables, is known to activate the Nrf2 pathway and have antioxidant and anti-inflammatory properties with potential anti-asthmatic effects^13,14^. Relaxin (RLN) is an anti-fibrotic hormone with pre-clinical potential in the OVA-induced chronic allergic airways models ^5,15^. Emerging evidence suggests that histone deacetylase inhibitors (HDACi), such as valproic acid (VPA) and trichostatin A (TSA), can modulate immune responses by skewing the Th1/Th2 balance and activating T-regulatory cell production^16^, as well as attenuating chronic allergic airways pathology such as reduced airway constriction induced by methacholine ^7^, decrease innate inflammatory markers ^17^ and Th2 response^18^. Evaluating the therapeutic benefits of these experimental compounds in murine OVA-induced models of chronic allergic airways disease are usually done using conventional methodologies such as morphometric histological assessment, immunohistochemistry, or respiratory measurements^10,19–22^. While this approach has been successful, elucidating detailed molecular mechanisms of action has proven more challenging.

Synchrotron Fourier-transform infrared (S-FTIR) microspectroscopy has been used to characterise the mechanism of action of novel therapeutics at the molecular level. The FTIR microspectroscopic technique has been utilised as a diagnostic tool for cancers such as liver^23^, lung^24^ and ovarian cancer^25^, and for kidney stone formation^26^. Furthermore, it has also been used to identify biochemical fingerprints of novel anti-asthmatic therapies such as leaf extracts from *Anacardium occidentale*^27^, cromolyn sodium^28^, and theophylline^29^, and a number of our experimental therapies of interest (Supplementary Table S1).

In this study, we utilised an analytical approach based on the high spatial resolution S-FTIR microspectroscopic technique and principal component analysis (PCA) to investigate the biochemical effects of experimental compounds, RV, VPA, TSA, LSF, and RLN in prevention and reversal models of OVA-induced chronic allergic airways disease. Due to the S-FTIR technique providing a superior spectral quality, in terms of signal to noise ratio, at diffraction-limited spatial resolution for mapping the airways, the time to acquire these maps can be significant. Therefore, we also employed the offline focal plane array–FTIR (FPA-FTIR) imaging technique for a fast acquisition of much larger chemical maps, to enable a broader molecular overview of the surrounding airway tissue. Overall, the aims were to identify key biochemical changes to provide further insights into their mechanism of action, and to correlate our findings with data previously collected in these models using conventional methodologies.

## Methods

### Animals

Six-week old female BALB/c mice were used to establish the prevention and reversal OVA-induced chronic allergic airways models. These studies complied with the Australian guidelines for the care and use of laboratory animals for scientific purposes and approved by the Monash University animal ethics committee (MARP/2012/085).

### Chronic allergic airways disease model

**Figure 1:**
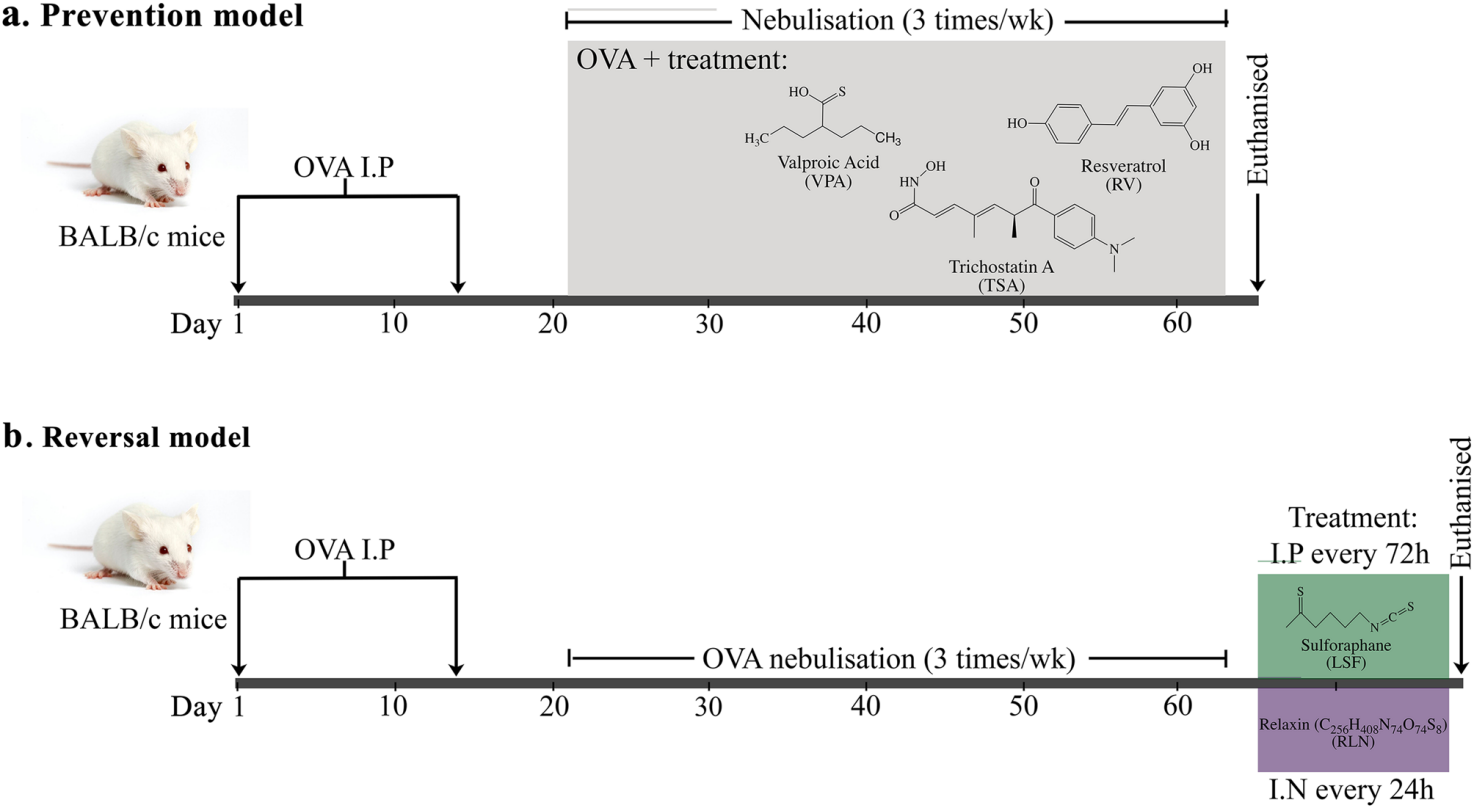
Two murine models of OVA-induced chronic allergic airways timelines. The difference between the prevention **(a)** and reversal **(b)** murine OVA-induced chronic allergic airways model timelines.

**Figure 2:**
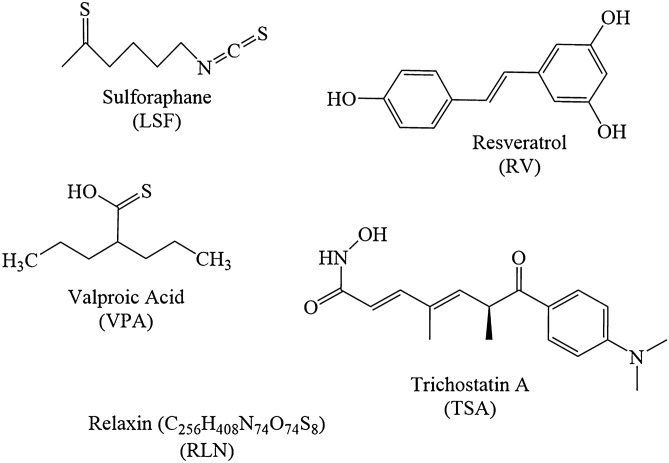
Chemical structure of antioxidant and chromatin modifying compounds.

The chronic allergic airways disease model has been previously described^20^. Briefly, control mice (*n* = 5) were sensitised with 500 µL of 0.9% saline solution intraperitoneally (IP) (Baxter Health care, New South Wales, Australia). The positive control mice (OVA; *n* = 5) and the mice in experimental groups were sensitised with Grade-V OVA (10 µg) (Sigma-Aldrich, MO, USA) with aluminium potassium sulphate adjuvant (400 µg) (AJAX Chemical, Kotara, New South Wales, Australia) diluted in 0.9% saline solution, on days 0 and day 14. Mice were nebulised with OVA for 30 min, three times a week for 6 weeks, from day 21 to day 63. Treatment groups were divided into the prevention and reversal models according to the timelines shown in Fig. 1, and chemical structures of compounds used in the experimental groups are shown in Fig. 2.

### Prevention model

Mice were administered (IP) with RV (12.5 mg/kg; *n* = 2) (Sigma-Aldrich), VPA (20 mg/ml; *n* = 2) (Sigma-Aldrich) or TSA (5 mg/kg; *n* = 2) (Sigma-Aldrich) three days per week for six weeks during the OVA nebulisation period, as previously described^20–22^. Mice were euthanised IP with ketamine and xylazine (200 µg/g: 10 µg/g) 4 days after the last nebulisation, on day 67. Lung tissues were removed, fixed with 10% buffered formalin for 24 h before paraffin embedding.

### Reversal model

Mice were treated with the indicated compounds on day 64, 24 h after the last OVA nebulisation period. This sub-group of mice were either administered (IP) with LSF (5 mg/kg; *n* = 3) (Sigma-Aldrich) five times every 72 h, or daily intranasal (IN) treatment with RLN (0.5 mg/kg/day; *n* = 3) (Corthera Inc., San Carlos, CA, USA) for 14 days, as previously described^10^. Mice were euthanised IP with ketamine and xylazine (200 µg/g: 10 µg/g) 24 h after the last treatment, on day 78. Lung tissue was removed, fixed with 10% buffered formalin for 24 h before paraffin embedding.

### Sample preparation for FTIR microspectroscopy and histological assessment

Lung samples were sectioned (4 µm in thickness) with a Leica RM 2135 microtome (Leica Biosystems, Wetzlar, Germany) onto either double frosted microscope slides (Mikro-Glass, Australia) for histological assessment or calcium fluoride (CaF_2_) windows (Crystan, Dorset, UK) for FTIR microspectroscopy. Samples for FTIR microspectroscopic experiments were deparrafinised by two consecutive 5 min washes with xylene (Sigma-Adrich, Australia), and stored in a desiccator for spectral data collection.

### Focal plane array-Fourier transform infrared (FPA-FTIR) microspectroscopy

All lung tissue sections were imaged using an offline FPA-FTIR microspectroscopic system (Bruker Optik GmbH, Ettlingen, Germany) at the Australian Synchrotron Infrared Microspectroscopy (IRM) Beamline as described earlier^30,31^. Briefly, FPA-FTIR images were acquired in transmission mode with the Bruker Hyperion 3000 FTIR microscope, with a liquid nitrogen cooled 64 × 64 element FPA detector and a matching 15 × objective and condenser (NA = 0.40) coupled to a Bruker Vertex 70 FTIR spectrometer with an internal thermal (Globar) IR source. Each FPA-FTIR image were acquired within the 4,000–800 cm^-1^ spectral region with a sampling area of 180 × 180 μm^2^.

The FPA-FTIR spectral images were acquired as previously described^30,31^. Briefly, spectral images were acquired with 8 cm^−1^ resolution, 64 co-added scans, Blackman-Harris 3-term apodisation, Power-Spectrum phase correction, and a zero-filling factor of 2 using the OPUS 7.2 imaging software (Bruker). Furthermore, background control measurements were taken using the same acquisition parameters, by focusing on a clean surface area of the CaF_2_ window without any tissue sample.

For the prevention model, 4 × 4 FPA grids were collected from the saline (*n* = 5), OVA (*n* = 6), RV (*n* = 1), TSA (*n* = 2) and VPA (*n* = 2) groups. For the reversal model, FPA-FTIR images were collected for the saline (*n* = 5), OVA (*n* = 5), LSF (*n* = 2), and RLN (*n* = 3) groups, with the grid sizes collected in either a 4 × 4 (*n* = 5) or 8 × 8 (*n* = 9).

Prior to analysis, all FPA-FTIR images were processed for an atmospheric water correction, followed by baseline correction, vector normalisation and second derivative (25 smoothing points). The spatial distribution of protein on the tissue samples were produced based on integrated areas under the amide I band (1,695–1,600 cm^−1^). A random selection of at least 50 spectra per image was extracted from the lung epithelium and surrounding tissue and averaged to create an absorbance and inverted second derivative spectra of all the samples.

### Synchrotron-FTIR microspectroscopy

The S-FTIR microspectroscopic measurement was performed at the Australian Synchrotron IRM Beamline as previously described^31,32^. Briefly, microspectroscopic measurements were taken in transmission mode using a Bruker Vertex 80v spectrometer coupled with a Hyperion 2000 FTIR microscope (Bruker Optik GmbH, Ettlingen, Germany) and a liquid nitrogen cooled narrow-band mercury cadmium telluride (MCT) detector. The S-FTIR measurement was collected with a 36 × IR objective and condenser (NA = 0.50; Bruker Optik GmbH, Ettlingen, Germany) and an aperture size of 5 µm in diameter for every pixel and for every step interval between pixels.

The spectral range of 3,800–700 cm^−1^ using 4 cm^−1^ spectral resolution and 16 co-added scans was utilised for each S-FTIR spectrum. A Blackman-Harris 3-term apodisation, Power-Spectrum phase correction and a zero-filling factor of 2 were utilised as the default acquisition parameters using the OPUS 7.2 software suite (Bruker).

S-FTIR spectral maps of one airway per mouse were acquired from the prevention model including saline (*n* = 2), OVA (*n* = 2) and RV (*n* = 2). For the reversal model, data was acquired for the saline (*n* = 4), OVA (*n* = 3), LSF (*n* = 3), and RLN (*n* = 3) groups, with one airway per mouse, with the exception of the saline and OVA groups which had, two airways mapped for two mice, and one mouse, respectively.

### Spectral pre-processing and multivariate data analysis

FTIR spectra were first quality-screened using CytoSpec v. 1.4.02 (Cytospec Inc., Boston, MA, USA), to extract and obtain good-quality spectra (> 250 spectra per tissue sample). These spectra were then imported into The Unscrambler 10.1 software package (CAMO Software AS, Oslo, Norway) for PCA. All spectra were transformed into second derivatives using 25-point Savitzky-Golay algorithm to eliminate the broad baseline offset and curvature^33^. The resultant derivative spectra were corrected by extended multiplicative scatter correction (EMSC) method^34^ based on biological bands in the spectral regions of 3,063–2,773 cm^−1^ and 1,790–1,018 cm^−1^. PCA was subsequently performed using the same spectral ranges on the combined datasets, in order to investigate similarities and differences between the tissue groups, and presented in forms of scores and loadings plots^35,36^.

### Curve fitting analysis

For a comparison of experimental techniques a curve fitting analysis was conducted on the reversal model for the LSF treated mice (*n* = 3 per group) S-FTIR maps, and compared to matching lung samples from the histological results of mean-sub-epithelial thickness to compare the different methods for detecting collagen deposition. Briefly, the second inverted second derivative was cut to the spectral region of 1,265–1,990 cm^−1^, then a new baseline was corrected to the lowest minimum peak of the spectra, and a curve fitting analysis was used based on a Lorentz and Gaussian shape with a maximum of three curves per spectra, to determine the integral of the collagen peak at 1,200 cm^−1^, using OPUS 7.2 software suite (Bruker).

### Statistical analysis

Statistical analysis was performed on the comparison of methods for collagen detection with GraphPad Software Prim v8.0 (San Diego, CA, USA). Results were analysed by a Pearson’s correlation analysis. Data was considered statistically significant with a p-value less than 0.05.

## Results

### FPA-FTIR microspectroscopy

**Figure 3:**
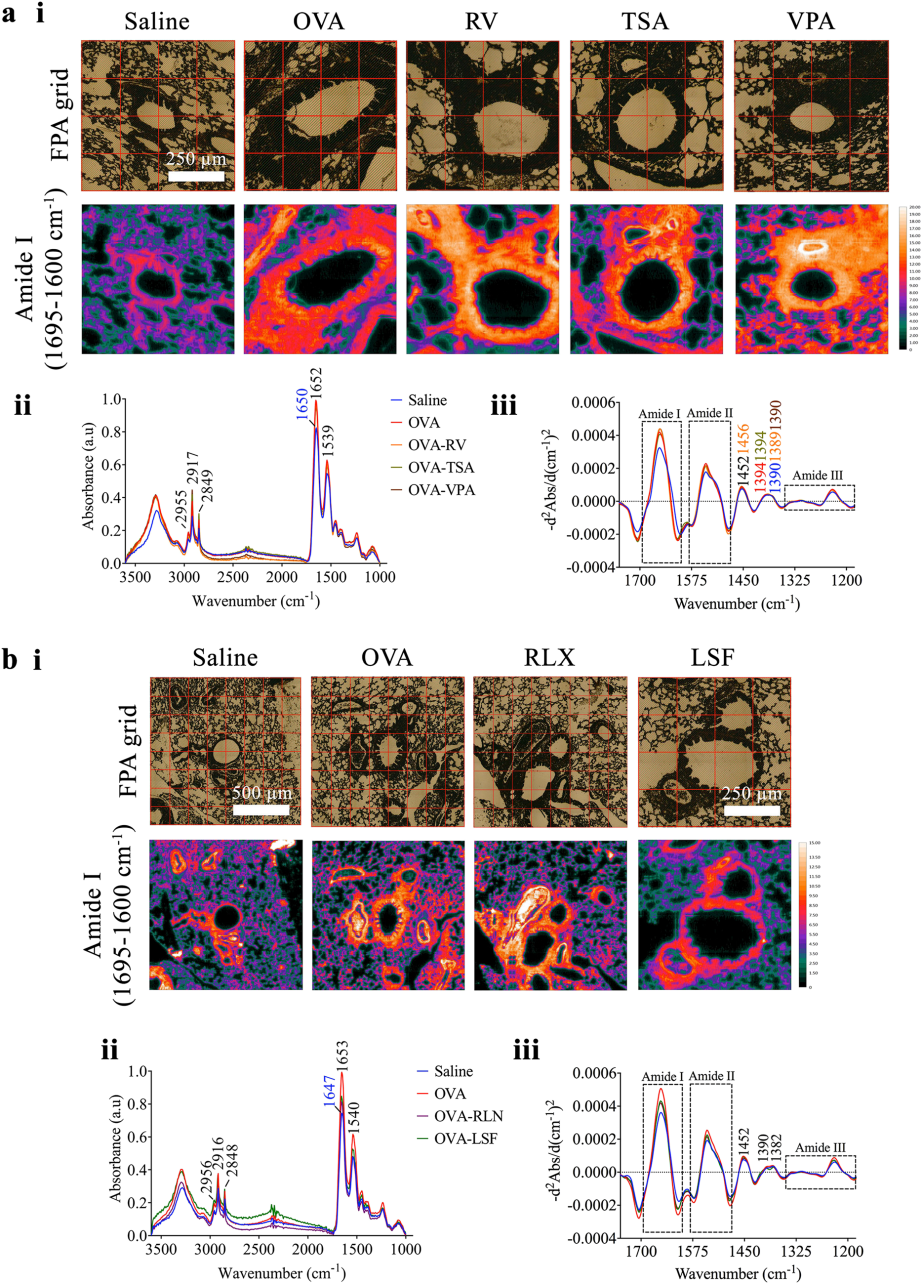
FPA-FTIR chemical images of protein distribution and average absorbance spectra obtained from the two models of allergic airways disease. The chemical images of the prevention model **(a)** and the reversal model **(b)** were produced based on integrated areas under inverted second derivate spectra for amide I band (1,695–1,600 cm^−1^) (i). A number of spectra were randomly selected from each image (> 50 spectra) to obtain the average absorbance (ii) and inverted second derivative (iii) spectra.

The offline FPA-FTIR microscope provides a general overview of the biochemical fingerprint within the tissue samples. It enables for faster acquisition of larger infrared maps to be taken compared to the higher spectral quality maps obtained from the online S-FTIR. The laboratory-based FPA-FTIR measurement was first performed on all the lung tissue samples to obtain molecular information, specifically the protein distribution, on a much larger area of tissue. The chemical images of the protein distribution based on the amide I band (Fig. 3a, i and b, i) display strong intensity of the protein components in the OVA airway compared to the saline group. In particular, all the treated groups in the prevention model (Fig. 3a, i) exhibit considerably stronger amide I intensities, particularly within the nucleus of epithelial cells, compared to the saline control group. In the reversal group, LSF resembles analogous amide I intensity to the saline control group, while RLN, shows an equivalent level of amide I intensity as those observed for the OVA group (Fig. 3b, i). Furthermore, the chemical image of the LSF group indicates substantially higher peak intensities at 2,956 and 2,872 cm^−1^, compared to all other treatment groups, indicative of protein acetylation^37^.

Comparing the average spectra (Fig. 3a, ii, and b, ii) and the inverted second derivative spectra (Fig. 3a, iii, and b, iii), the position and shape of the amide I and II bands closely resemble the corresponding bands in the saline control groups, suggesting no change in the combination of protein secondary structures in the treated groups.

### Synchrotron-FTIR microspectroscopy

The high brightness of the S-FTIR technique results in an enhanced spectral quality in terms of signal to noise (S/N) ratio, which enables acquisition of high-quality FTIR spectra at diffraction-limited spatial resolution. High-quality spectra plays an important role in the accuracy of the subsequent multivariate data analysis results. A summary of the key peaks identified in the S-FTIR results is reported in Table 1.

**Table 1:** Summary of key peaks and their corresponding band assignment.

Wavenumber (cm^−1^)	Band assignment
2,872 & 2,960	CH_3_ acetylation
2,849 & 2,917	CH_2_ propionylation
1,695–1,600	Amide I
1,543	Amide II
1,456	*δ*_as_(CH_3_) and *δ*(CH_2_) vibrations
1,350–1,180	Amide III region
1,379	*δ*(CH_3_)
1,293	Either N–H thymine, of deformation of N–H cytosine
1,236	PO_2_^−^ asymmetric stretching of nucleic acids
1,180	CH_2_ stretching
1,200	Collagen
1,088	ν_s_(PO_2_^−^) stretching

#### (i) Absorbance spectrum

**Figure 4:**
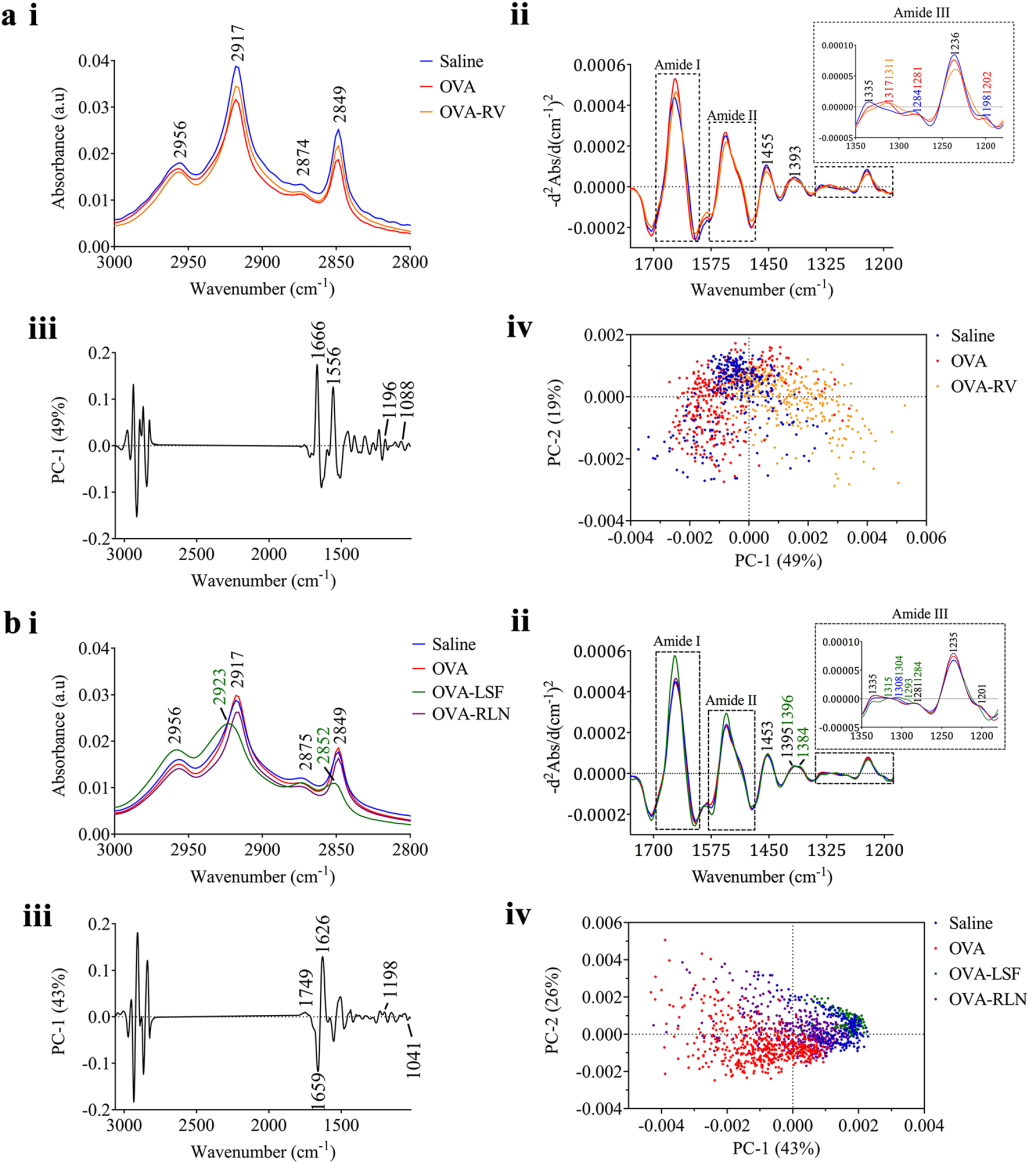
Average S-FTIR spectra and the corresponding PCA analysis obtained from the two models of allergic airways disease. PCA analysis based on S-FTIR spectra of the prevention **(a)** and reversal **(b)** models. The average absorbance (i) and inverted second derivative (ii) spectra obtained from each treatment group, accompanied by the resultant PCA analysis including loadings (iii) and scores (iv) plots.

S-FTIR absorbance spectra observed for the prevention (Fig. 4a, i) and reversal (Fig. 4b, i) models. In the prevention model (Fig. 4a, i), no major variances amongst the experimental groups were observed at the protein acetylation (2,827 and 2,960 cm^−1^) and propionylation (2,851 and 2,922 cm^−1^) bands. In the reversal model (Fig. 4b, i), LSF displays highest peak intensity at 2,956 cm^−1^ compared to all the other groups, this was also confirmed in the FPA-FTIR results, however LSF displayed a lower peak intensity than the saline group at 2,875 cm^−1^. An increase in these peaks 2,956 and 2,875 cm^−1^ was observed, indicative of CH_3_ protein acetylation. The LSF treated group also displayed a strong shift to higher wavelength and decrease in band intensity at peaks 2,923 and 2,852 cm^−1^, indicative of CH_2_ protein propionylation.

#### (ii) Inverted second derivative spectrum

The inverted second derivative spectra reveal subtle peak changes in the spectral features that are not observed on the absorbance spectra. Here, we specifically focused to the amide region (1,700—1,200 cm^−1^) and further into the amide III region (1,350–1,200 cm^−1^) which is important for investigating changes in the protein secondary structures.

Within the amide III region of the prevention model (Fig. 4a, ii), the saline group exhibited a peak at 1,335 cm^−1^, representing α-helical structures typical of type I collagen^38,39^. The OVA and RV group, on the other hand, showed peaks at 1,317, and 1,311 cm^−1^, respectively, indicative of β-sheets formations^38^. Random coil structures at band peak 1,284 cm^−1^^38^ are observed in the saline and OVA groups, although the OVA group has a decrease in peak intensity. Further, no random coil structures are observed at the 1,284 cm^−1^ for the RV group, or a peak at 1,200 cm^−1^, which is attributed to collagen^40^.

In the reversal model (Fig. 4b, ii), at the band centered at ~ 1,393 cm^−1^, typical of CH_2_ asymmetric bending and COO– stretching of proteins and fatty acids^41^, LSF displays a broadening of this band into two peaks, at 1,396 and 1,384 cm^−1^, which correspond to CH_3_ deformation, and stretching of C–O and the deformation of rather C–H and N–H bonds, respectively^42–44^. While, in the amide III region of the reversal model (Fig. 4b, ii), the OVA and RLN treated groups revealed an increase in α-helical structures, while the saline and LSF treated groups had an increase in β-sheet and random coil structures^38^. Further, the LSF group did not have a peak at 1,201 cm^−1^.

#### (iii) Principal component analysis

The scores plot in the prevention model displays clear clusters amongst the three experimental groups (Fig. 4a, iv). The initial PCA result displays a representative of one mouse from each experimental group, as each experimental group demonstrated consistency between individual mice (Supplementary Fig. S2). The saline and OVA groups are largely clustered on the negative side of the PC-1 axis, whereas the RV group is clustered further away from the saline control group on the positive side of the PC-1 axis. Since the separation occurred along the PC-1 axis, the difference in molecular composition between these three groups can be examined through the PC-1 loadings plot. The PC-1 loadings (Fig. 4a, iii), which correlates to 49% of the variance, revealed strong negative loadings at 1666 and 1556 cm^−1^, which correspond to amide I and II bands, respectively, indicating that the major change in the molecular structure among these three groups is due to the protein components. There are also small contributions coming from the minor loadings at 1,196, and 1,088 cm^−1^, which could be attributed to collagen, and nucleic acid PO_2_^-^, respectively.

Similarly, the reversal model displays a clear clustering feature amongst all experimental groups in the scores plot (Fig. 4b, iv). Most groups are clustered within the positive side of the PC-1 axis in the scores plot. The variance along the PC-1 axis (43%) (Fig. 4b, iii) is largely attributed to heavily loaded peaks at 1659 and 1626 cm^−1^, suggesting that the difference in protein conformation is the major cause of the separation. While the saline, LSF- and RLN-treated groups presented proportionally more of protein in α-helical structure (according to the positive loading at 1659 cm^−1^), the OVA group is likely to possess protein in β-sheet conformation (negative loading at 1626 cm^−1^) with a small contribution from collagen component (1,198 cm^−1^). Furthermore, variance along the PC-2 axis (26%) displays a strong separation between the OVA-LSF and OVA treated groups. The major peaks causing this separation are the same peaks causing the separation along the PC-1 axis, as mentioned above. Therefore, this highlights the differences amongst the OVA and OVA-LSF treated groups is amplified due to these peaks, along both the PC-1 and PC-2 loadings.

The key difference observed amongst the prevention and reversal models is the presence of peak 1556 cm^−1^, as seen in the prevention model loadings plot (Fig. 4a, iii). This band is attributed to α-helices and anti-parallel β-sheets^45^.

### Comparison between S-FTIR and histology methods for measurement of collagen deposition

**Figure 5:**
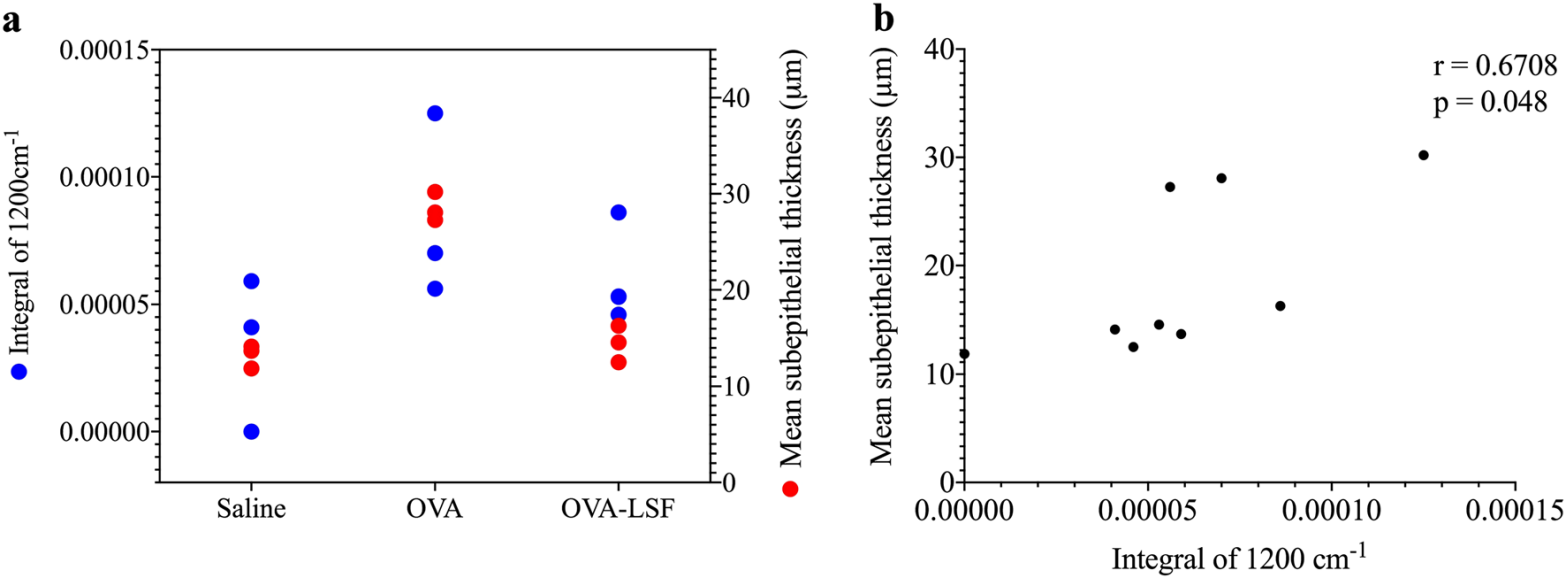
Comparison of collagen presented in the reversal model of allergic airways disease, based on the data obtained from histology (red) and S-FTIR spectra (blue). The mean subepithelial thickness calculated from a Masson’s trichrome stain and the integration of band 1,200 cm^−1^ calculated from a curve fitting analysis were used to identify collagen deposition in each method and are plotted together for comparison purposes **(a)**. A Pearson’s correlation was performed on the two methods **(b)** and found to have a statistically significant (p = 0.0479) positive correlation (r = 0.6708).

A consistent pattern was observed overall between the two methods for collagen detection (Fig. 5). The observed integral of 1,200 cm^−1^, calculated from a curve fitting analysis, displayed an increased collagen deposition within the OVA control group, consistent with previous observations^20–22^, compared to both the saline and LSF treated group, for both conventional histology and S-FTIR methods (Fig. 5a). A Pearson’s correlation (Fig. 5b) found a positive correlation (r = 0.6708) amongst the two methods and was found to be statistically significant (p = 0.048).

## Discussion

A range of experimental compounds RV, LSF, RLN, VPA and TSA have demonstrated their anti-asthmatic therapeutic potential in well-established chronic allergic airways models^10,19–22^. However, their mechanistic effects remain challenging to distinguish through conventional methods. Henceforth in this study we utilised a novel analytical approach of FPA-FTIR and S-FTIR microspectroscopy to investigate further insights into their mechanism of action, as well as correlate, where possible, these findings to conventional methods.

The S-FTIR microspectroscopy provided us with a better understanding of the key biochemical changes as a result of the different treatments in two variations of the OVA-induced chronic allergic airways murine model. The integration of amide I band, after pre-processing and normalisation of the tissue, highlights that in the prevention model, RV, TSA and VPA led to increased intensities of the amide I band, suggesting an enhanced proportion of protein composition particularly those localised within the nucleus region of epithelial cells^43^. However, it remains unclear why the same was not observed in the reversal model with the LSF treated group compared to the controls.

Previous studies have investigated HDACi in malignant tissue samples using FTIR microspectroscopy to characterise protein acetylation^37,46^. Acetylation is commonly attributed to alterations of CH_3_ stretching vibration at 2,872 and 2,960 cm^−1^ and the key band indicative of CH_2_ propionylation can also be observed based on the peaks 2,851 and 2,922 cm^−1^. Our findings indicate an increase in band intensities of the CH_3_ stretching band at 2,956 cm^−1^ observed for the LSF treated group, in both the FPA-FTIR and the S-FTIR data, as well as a decrease in the associated CH_2_ propionylation bands. One explanation for this effect is likely due to the known chromatin modifying properties of LSF, specifically as an HDACi^47,48^. Our finding are in agreement with Chen and co-workers attributing the intensity and alterations of the CH_3_ band to acetylation in their study of HDACi SNDX-275 treated cancer patients in serum samples^37^. However, our prevention model did not indicate an increase in CH_3_ stretching band in neither the FPA-FTIR nor S-FTIR data. While RV is a known HDACi^11^, factors such as the dose and model used (prevention model) may have had a differential effect on protein acetylation compared to the reversal model. Similarly, Singh and co-workers found that sodium butyrate did not show alterations on the CH_3_ acetylation bands, even though it is a known HDACi^46^.

The OVA-allergen utilised in our models plays a crucial role in inducing irreversible airway remodeling, a hallmark characteristic of chronic allergic airways disease^21,49,50^. It produces increased epithelial wall thickening, which contributes to airway obstruction and fibrosis and increased collagen deposition surrounding the airways^51–53^. The type of collagen deposition seen in human asthma is largely type I and III, and varies depending on disease severity^54^. Using standard morphometric techniques, some of our previous studies and others have demonstrated that RV, TSA, VPA, RLN and LSF reduced collagen deposition in the airway walls^10,19–22^. However, this approach was unable to differentiate the type of collagen that is modulated by these compounds. Picrosirius red stain can detect the type of collagen deposition but is not routinely measured.

In this study we confirmed that the peak at ~ 1,200 cm^−1^ correlates with collagen deposition, as compared to conventional sub-epithelial thickness measurements, validating this approach. Previous reports have identified characteristic peaks for collagen in biological samples using S-FTIR microspectroscopy (Supplementary Table S3). Both the RV and LSF treated samples showed an analogous absence of the peak at 1,200 cm^−1^ to the saline healthy control group, whereas the OVA group indicated the presence of this peak. Furthermore, in our comparison of S-FTIR at the band 1,200 cm^−1^ and histological staining techniques, our results displayed a positive correlation amongst the two techniques. This suggests the potential application of the S-FTIR technique for semi-quantitative analysis of collagen deposition in the lung tissue with a good correlation to the conventional histological approach. However, it is important to note that the band assignment of 1,200 cm^−1^ is not specific to the type of collagen present. For the S-FTIR spectra, the type of collagen is typically identified in the amide III region, based on changes in protein secondary structures. Our results suggested type I collagen, typically attributed to α-helical structures at 1,336 cm^−1^^55^, which was increased in the OVA groups, and decreased in the saline and LSF groups. However, this peak may also be attributed to other α-helical-like structures such as heat shock proteins which are also known to be increased in asthma^56^. Furthermore, the saline and LSF treated groups, showed an increase in type III collagen deposition, characterized by an increase in β-sheet formations and random coil structures^57^. Our study indicates the sensitivity of S-FTIR to accurately detect collagen deposition in a similar manner to conventional histology. S-FTIR has the advantage of allowing specific identification the type of collagen.

Allergic inflammation is another major factor that contributes to irreversible remodelling of airway walls and increased bronchial hyperresponsiveness in people with asthma^58–60^. Using conventional methodologies, inflammation is characterised via histological assessment of immune cells infiltrating into airway tissues, changes to the composition of immune cells in bronchoalveolar lavage fluid, and key changes in inflammatory markers^61^. Several reports have identified inflammation by biochemical changes observed through the S-FTIR spectra^62–64^. Based on our data, however, specific peaks characteristic of inflammation were not observed in the acquired S-FTIR spectra. This could be due to the nature of the high spatial resolution of S-FTIR microspectroscopy to insufficiently detect localised inflammation as compared to conventional histology.

It should be emphasized that there were a few peaks presented in the S-FTIR spectra, which are not typically observed through conventional FTIR techniques. These include the peaks at 1,396 and 1,384 cm^−1^, which were found only in LSF treated group, whereby these peaks were reported to be largely indicative of symmetric CH_3_ and C–O stretches, as well as C–H and N–H deformation^65,66^. Furthermore, the peak at 1,236 cm^−1^, representative of asymmetric stretching vibration of PO^2−^ from nucleic acids^67^ was decreased in RV and LSF groups. However, the exact significance of these peaks in the context of chronic allergic airways is yet to be elucidated. These characteristic peaks specific to the S-FTIR microspectroscopic technique may provide further information into their mechanistic actions as potential anti-allergic therapeutics, although will need to be confirmed through conventional techniques.

One of the limitations to the S-FTIR microspectroscopic technique is that the DNA region (below 1,000 cm^−1^) cannot be detected with the IR spectrum using calcium fluoride transmission windows. This region is of interest, particularly when considering chromatin-modifying compounds, and in future studies alternative transmission windows such as barium fluoride windows could be used to mitigate this limitation. Nevertheless, the technique has provided novel insights into the biochemical changes, independent to the DNA region.

## Conclusion

Overall, the application of S-FTIR in combination with offline FPA-FTIR imaging has provided a better understanding of the biochemical changes within complex tissue samples taken from two models of chronic allergic airways disease. The S-FTIR technique enabled detection of changes in collagen deposition, nucleic acids, and protein acetylation. Although these bands may be identified through conventional methodologies including histological assessment and immunofluorescence studies, S-FTIR technique offers an approach to collectively identify these changes in individual maps, with minimal sample preparation required. Further, using S-FTIR numerous novel peaks associated with the experimental compounds were identified. It will be interesting to clarify if these novel peaks are biologically important and mechanistically contribute to potential anti-allergic effects.

## Supplementary information

Supplementary Information. (PDF 431 kb)
