# Supplementary material for: Investigation of molecular mechanisms of experimental compounds in murine models of chronic allergic airways disease using synchrotron Fourier-transform infrared microspectroscopy
Source: Sci Rep. 2020 Jul 16;10:11713. doi: 10.1038/s41598-020-68671-2 (PMC7366655; doi:10.1038/s41598-020-68671-2)
Supplement: Supplementary file 1 — Supplementary Information. (PDF 431 kb) [file 41598_2020_68671_MOESM1_ESM.pdf]

Supplementary Information

Investigation of molecular mechanisms of experimental compounds in murine models of chronic allergic airways disease using synchrotron Fourier-transform infrared microspectroscopy

Nadia Mazarakis[1,2,3], Jitraporn Vongsvivut[4], Keith R Bambery[4], Katherine Ververis[1], Mark J Tobin[4], Simon G Royce[6], Chrishan S Samuel[6], Kenneth J Snibson[2], Paul V Licciardi[3,5], Tom C Karagiannis[1,7,]*

[1] Epigenomic Medicine Laboratory, Department of Diabetes, Central Clinical School, Monash University, Alfred Centre, Melbourne, Victoria 3004, Australia

[2] Faculty of Veterinary and Agricultural Sciences, University of Melbourne, Parkville, Victoria 3010, Australia

[3] Murdoch Children's Research Institute, Melbourne, Victoria 3004, Australia

[4] ANSTO Australian Synchrotron, Clayton, Victoria 3168, Australia

[5] Department of Paediatrics, University of Melbourne, Parkville, Victoria 3010, Australia

[6] Monash Biomedicine Discovery Institute and Department of Pharmacology, Monash University, Clayton, Victoria 3168, Australia

[7] Department of Clinical Pathology, University of Melbourne, Parkville, Victoria 3010, Australia

Author for correspondence:

*Tom C Karagiannis
Epigenomic Medicine Laboratory

Department of Diabetes, Central Clinical School, Monash University
Alfred Centre, 99 Commercial Road, Melbourne, Victoria, 3004, Australia
Email: tom.karagiannis@monash.edu
Phone: +61 3 9903 0491

Supplementary Figures and Tables

Supplementary Table S1. FTIR band assignment of functional groups commonly found in antioxidant and chromatin modifying compounds.

**Compound**	**Wavenumber (cm^{-1})**	**Band assignment**	**Significance**	**References**
Valproic acid				
	2872 and 2960	Acetylation (CH_3 stretching of proteins and lipids, respectively)		[1]
	2851 and 2922	CH_2 stretching (of proteins and lipids respectively) – posttranslational protein modifications	VPA treated U343 glioma cells.<br>Increased intensity of VPA treated cells, as compared to control.<br>Specifically HDACi increase CH_2 band intensity more directly than CH_3, due to its inhibitory effects on propionyl CoA synthase.	[2]
	~2992-2850	$v_{as}(CH_3)$ and $v_s(CH_3)$ vibrations	Decreased with increased dose of VPA in HeLa cells	[3]
Trichostatin A				
	930	Z-form DNA increased significantly	Acetylation of lysine residues on B-DNA, caused increase left-handed Z-DNA formation, with increased TSA dose in HeLa cells.	[4]
Resveratrol				
	1633	Amide I	Shift from 1638 to 1633cm^{-1} of secondary structural changes with the addition of	[5]

			RSV to human serum albumin.	
	1656 and 1545	Amide I and II	Absorbance intensity at the amide I band increased in BSA with RSV (0.125mM) in the difference spectra (RSV-BSA)	[6]
	1658	Amide I	Absorbance intensity at the amide I band decreased in BSA with RSV (0.5mM) in the difference spectra (RSV-BSA)	
Sulforaphane				
	1714 to 1710 and 1700	Guanine band	Calf thymus DNA solution with LSF (r= 1/40 and r=1/5, respectively)	[7]
	1655 to 1661			
	1610 to 1614	Thymine band	LSF (r=1/40 and 1/5)	
	1226 to 1230			
		Adenine band	LSF (r=1/5)	
		Phosphate band	LSF (r=1/5), which was only observed in higher concentrations of LSF	

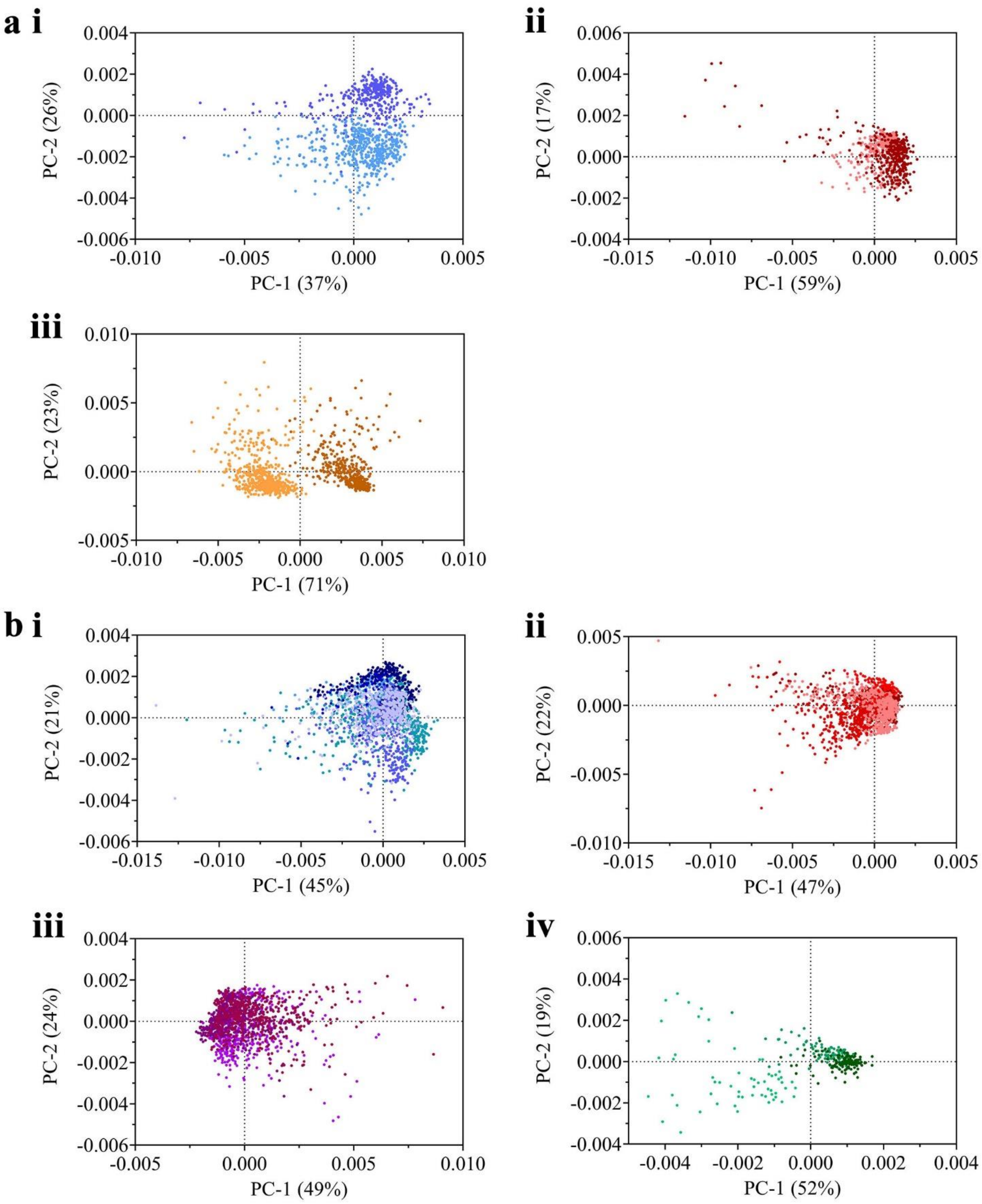

Supplementary Figure S2. Reproducibility of S-FTIR scores plots taken for each treatment group within each model. The treated groups from the prevention model (a); saline (*n*=2) (i), OVA (*n*=2) (ii), and RSV (*n*=2) (iii). The reversal model (b) treatment groups; saline (*n*=4) (i), OVA (*n*=3) (ii), RLN (*n*=3) (iii), and LSF (*n*=3) (iv).

Supplementary Table S3. FTIR band assignment of functional groups commonly found in chronic allergic airways diseases.

Wavenumber (cm^{-1})	Band assignment	References
1010-1020	Z-form DNA	[4]
1029, 1080, and 1150	Glycogen	[8]
1080 and 1240	PO^{-2} asymmetric and symmetric bonding of nucleic acids	[2]
1044-1069	Z-form DNA	[4]
1059	Nucleic acids (backbone of A-DNA)	[9]
1089	B-form DNA	[4]
1119	Nucleic acids. Stretching mode of the ribose C−O group in RNA	[10]
1124	PO_3^{-1} asymmetric stretching vibrations from DNA, RNA and phospholipids	[11]
1158–1173	v_{as}(CO-O-C) vibration of cholesterol esters	[12]
1165	Glycogen	[13]
1200	Collagen	[14]
1206, 1235 and 1280	Triplet protein band	[15]
1236	Amide III	[2]
1238	$v_{as}(PO_2^-)$ mode is the main marker of A-DNA conformation	[9]
1280	Random coil structures	[16]
1312	β-sheets, or α-helical structures	[16]
1312–1340	Amide III	[15]
1336	α-helical collagen	[17]
1376	CH_2 wagging vibrations of tyrosine	[11]
1383–1395	v_s(COO) stretching mode of the side chains of amino acid residues	[15]
1399	CH_3 deformation	[18]
1451	CH_2 deformation	[18]
1456	CH_2 bending mode of lipids	[12]
1514	Side chain resides of tyrosine	[19]
1560	Amide II, C-N stretching	[11]

1623–1637	β-plated sheet	[15]
1635	β-sheet secondary structure of amide I	[20]
1637	O–H bonding	[21]
1655	α-helix	
1657	α-helical structure of amide I	[22]
1670–1695	β-turns and anti-parallel β-plated sheets	
1686	Unordered, random coils and turns	[23]
1740	Phospholipids due to ester groups [ν(C–O)]	
1745	(ν(C=O) carbonyl stretching band of triglycerides and cholesterol esters	[12]
2850	Lipid synthesis and/or membrane structure	
2851	CH_2 group stretching in protein (methylene)	[2]
2853	Lipids [$\nu_s(CH_2)$]	
2872	C-H asymmetric and symmetric stretching of methyl (CH_3) groups in proteins	[1]
2923	Lipid synthesis and/or membrane structure	[24]
2922	Anti-symmetric stretching of CH_2 group stretching in lipids (methylene)	[2]
2925	Lipids [$\nu_{as}(CH_2)$]	
2960	C-H asymmetric and symmetric stretching of methyl (CH_3) groups in lipids	[1]
3290	Amide A	[18]
3402	O–H bonding	[21]

References

1. Chen, T., et al., *Pharmacodynamic assessment of histone deacetylase inhibitors: infrared vibrational spectroscopic imaging of protein acetylation.* Anal Chem, 2008. **80**(16): p. 6390-6.
2. Singh, B., et al., *Fourier transform infrared microspectroscopy identifies protein propionylation in histone deacetylase inhibitor treated glioma cells.* J Biophotonics, 2012. **5**(3): p. 230-9.

3. Veronezi, G.M., et al., *DNA Methylation Changes in Valproic Acid-Treated HeLa Cells as Assessed by Image Analysis, Immunofluorescence and Vibrational Microspectroscopy.* PLoS One, 2017. **12**(1): p. e0170740.
4. Zhang, F., et al., *Histone Acetylation Induced Transformation of B-DNA to Z-DNA in Cells Probed through FT-IR Spectroscopy.* Anal Chem, 2016. **88**(8): p. 4179-82.
5. Nair, M.S., *Spectroscopic study on the interaction of resveratrol and pterostilbene with human serum albumin.* J Photochem Photobiol B, 2015. **149**: p. 58-67.
6. Bourassa, P., et al., *Resveratrol, genistein, and curcumin bind bovine serum albumin.* J Phys Chem B, 2010. **114**(9): p. 3348-54.
7. Abassi Joozdani, F., et al., *Interaction of sulforaphane with DNA and RNA.* PLoS One, 2015. **10**(6): p. e0127541.
8. Saravanakumar, M., et al., *Molecular metabolic fingerprinting approach to investigate the effects of borneol on metabolic alterations in the liver of nitric oxide deficient hypertensive rats.* Mol Cell Biochem, 2012. **362**(1-2): p. 203-9.
9. Whelan, D.R., et al., *Monitoring the reversible B to A-like transition of DNA in eukaryotic cells using Fourier transform infrared spectroscopy.* Nucleic Acids Res, 2011. **39**(13): p. 5439-48.
10. Wood, B.R., et al., *Shedding new light on the molecular architecture of oocytes using a combination of synchrotron Fourier transform-infrared and Raman spectroscopic mapping.* Anal Chem, 2008. **80**(23): p. 9065-72.
11. Kaznowska, E., et al., *The classification of lung cancers and their degree of malignancy by FTIR, PCA-LDA analysis, and a physics-based computational model.* Talanta, 2018. **186**: p. 337-345.
12. Liyanage, S., et al., *Optimization and validation of cryostat temperature conditions for trans-reflectance mode FTIR microspectroscopic imaging of biological tissues.* MethodsX, 2017. **4**: p. 118-127.
13. Eto, M., et al., *Glycogen synthase kinase-3 mediates endothelial cell activation by tumor necrosis factor-alpha.* Circulation, 2005. **112**(9): p. 1316-22.
14. Fujioka, N., et al., *Discrimination between normal and malignant human gastric tissues by Fourier transform infrared spectroscopy.* Cancer Detect Prev, 2004. **28**(1): p. 32-6.
15. Zohdi, V., et al., *Importance of tissue preparation methods in FTIR micro-spectroscopical analysis of biological tissues: 'traps for new users'.* PLoS One, 2015. **10**(2): p. e0116491.

16. Cai, S. and B.R. Singh, *A distinct utility of the amide III infrared band for secondary structure estimation of aqueous protein solutions using partial least squares methods.* Biochemistry, 2004. **43**(9): p. 2541-9.
17. Andrus, P.G. and R.D. Strickland, *Cancer grading by Fourier transform infrared spectroscopy.* Biospectroscopy, 1998. **4**(1): p. 37-46.
18. Wang, X., et al., *FTIR spectroscopic comparison of serum from lung cancer patients and healthy persons.* Spectrochim Acta A Mol Biomol Spectrosc, 2014. **122**: p. 193-7.
19. Zhang, J., et al., *Characterization of postmortem biochemical changes in rabbit plasma using ATR-FTIR combined with chemometrics: A preliminary study.* Spectrochim Acta A Mol Biomol Spectrosc, 2017. **173**: p. 733-739.
20. Baltacioglu, H., et al., *Effect of thermal treatment on secondary structure and conformational change of mushroom polyphenol oxidase (PPO) as food quality related enzyme: A FTIR study.* Food Chem, 2015. **187**: p. 263-9.
21. Tabish, T.A., et al., *Investigating the bioavailability of graphene quantum dots in lung tissues via Fourier transform infrared spectroscopy.* Interface Focus, 2018. **8**(3): p. 20170054.
22. Ami, D., et al., *Structural analysis of protein inclusion bodies by Fourier transform infrared microspectroscopy.* Biochim Biophys Acta, 2006. **1764**(4): p. 793-9.
23. Miller, L.M., M.W. Bourassa, and R.J. Smith, *FTIR spectroscopic imaging of protein aggregation in living cells.* Biochim Biophys Acta, 2013. **1828**(10): p. 2339-46.
24. Wiercigroch, E., et al., *FT-IR Spectroscopic Imaging of Endothelial Cells Response to Tumor Necrosis Factor-alpha: To Follow Markers of Inflammation Using Standard and High-Magnification Resolution.* Anal Chem, 2018. **90**(6): p. 3727-3736.
